# Temporary Bowel Ligation and Negative Pressure Therapy in Damage Control Surgery for the Management of Septic Shock Secondary to Non-traumatic Intestinal Perforation

**DOI:** 10.7759/cureus.84374

**Published:** 2025-05-18

**Authors:** Lourdes Camacho Ramirez, Catalina Ortiz Monasterio, Domingo J Coutinho Thomas, Luis Navarro, Enrique Jean Silver, Pablo Orozco Obregón

**Affiliations:** 1 Surgery, American British Cowdray Medical Center, Mexico City, MEX; 2 Medicine, Tecnológico de Monterrey, Mexico City, MEX; 3 Medicine, Escuela de Medicina y Ciencias de la Salud, Mexico City, MEX

**Keywords:** abdominal sepsis, negative wound pressure therapy (npwt), non-traumatic intestinal perforation, septic shock (ss), temporary bowel ligation

## Abstract

Abdominal sepsis from non-traumatic intestinal perforations in patients with multiple comorbidities presents a significant surgical challenge. The need for rapid intervention to manage contamination and restore intestinal continuity is often complicated by hemodynamic instability. A 64-year-old female with a history of cervical cancer and right colon adenocarcinoma in remission presented with severe abdominal pain, nausea, vomiting, and signs of septic shock. Imaging revealed dilated intestinal loops, thickened walls, free fluid, and signs of mesenteric congestion. An exploratory laparotomy revealed intestinal ischemia, extensive intra-abdominal contamination, and perforations. Temporary bowel ligation was performed, and negative pressure wound therapy (NPWT) using the ABThera™ system was applied to manage the open abdomen. After hemodynamic stabilization and improvement in laboratory values, a second-look procedure was performed, during which a delayed anastomosis was successfully completed. This case highlights the effective use of damage control surgery (DCS), temporary bowel ligation, and NPWT in complex abdominal sepsis management. Negative pressure wound therapy (NPT) facilitated contamination control, reduced intestinal edema, and prepared the patient for safe anastomosis in a later surgery. Although controversial, the decision to delay the anastomosis was advantageous in this case, as it minimized the need for a permanent stoma and allowed for the restoration of intestinal continuity. This report contributes to the growing body of evidence supporting the combination of temporary bowel ligation and NPT in the management of severe abdominal sepsis. It emphasizes the need for individualized treatment strategies, and further studies are required to establish clear patient selection criteria.

## Introduction

Intestinal perforation, particularly in patients with significant comorbidities and intra-abdominal sepsis, presents a highly complex surgical challenge. The need for rapid and effective interventions to control contamination and restore intestinal continuity often conflicts with the hemodynamic instability commonly observed in these patients, limiting the use of conventional therapeutic options.

Damage control surgery (DCS) has proven valuable for stabilizing critically ill patients, allowing definitive reconstruction to be deferred until physiological conditions improve. Temporary bowel ligation has been proposed as a useful maneuver to minimize initial surgical time and control intra-abdominal contamination [1]. Concurrently, negative pressure therapy (NPT) has become the gold standard for managing the open abdomen, facilitating fascial closure, preventing infections, and improving conditions for subsequent interventions [2].

This case report details the successful management of a patient with septic shock of abdominal origin secondary to bowel obstruction, utilizing a DCS strategy that included temporary bowel ligation and NPT. This case contributes to the medical literature by providing clinical evidence on the feasibility and utility of this strategy in a high-complexity scenario.

## Case presentation

A 64-year-old female patient with a history of cervical cancer treated with chemotherapy and routine endometrial surveillance, and right colon adenocarcinoma treated with laparoscopic hemicolectomy and ileotransversoanastomosis, both in remission, presented with severe abdominal pain, nausea, and vomiting. The abdomen was distended. Initial laboratory values included hemoglobin of 13.6 g/dL, white blood cell count of 6.55 × 10³/μL, lactate of 1.34 mmol/L, creatinine of 1.39 mg/dL, albumin of 3.3 g/dL, C-reactive protein of 51.57 mg/dL, and pH of 7.46. A detailed summary of laboratory evolution during hospitalization is presented in Table 1. Abdominal tomography revealed dilated bowel loops with wall thickening, mucosal enhancement, hydro-aeric levels, free fluid, air bubbles, and mesenteric congestion. A nasogastric tube was inserted, and the patient was optimized for surgical management.

**Table 1:** Laboratory results of the patient during hospitalization Trends in laboratory parameters from admission to discharge. Elevated inflammatory markers (CRP, WBC) and decreasing hemoglobin were observed postoperatively. A gradual normalization trend was seen over the course of hospitalization.

Laboratory Test (Reference Range)	11/04/23 (Admission)	12/04/23	13/04/23	14/04/23	17/04/23	27/04/23 (Discharge)
White Blood Cells (4.8–11.0 × 10³/μL)	6.55	9.03	16.34	12.79	13.64	7.04
Hemoglobin (13.5–16.5 g/dL)	13.6	12.0	11.0	9.9	10.0	9.8
C-Reactive Protein (0–0.50 mg/dL)	51.57	34.38	37.84	21.43	5.92	8.31
Lactate (0.5–2.0 mmol/L)	1.34	—	—	—	—	—
Albumin (3.97–4.94 g/dL)	3.30	2.92	2.72	2.58	2.38	2.61
pH (7.38–7.46)	7.46	—	—	—	—	—
Creatinine (0.50–0.90 mg/dL)	1.39	1.06	1.16	1.09	0.59	0.51

**Figure 1:**
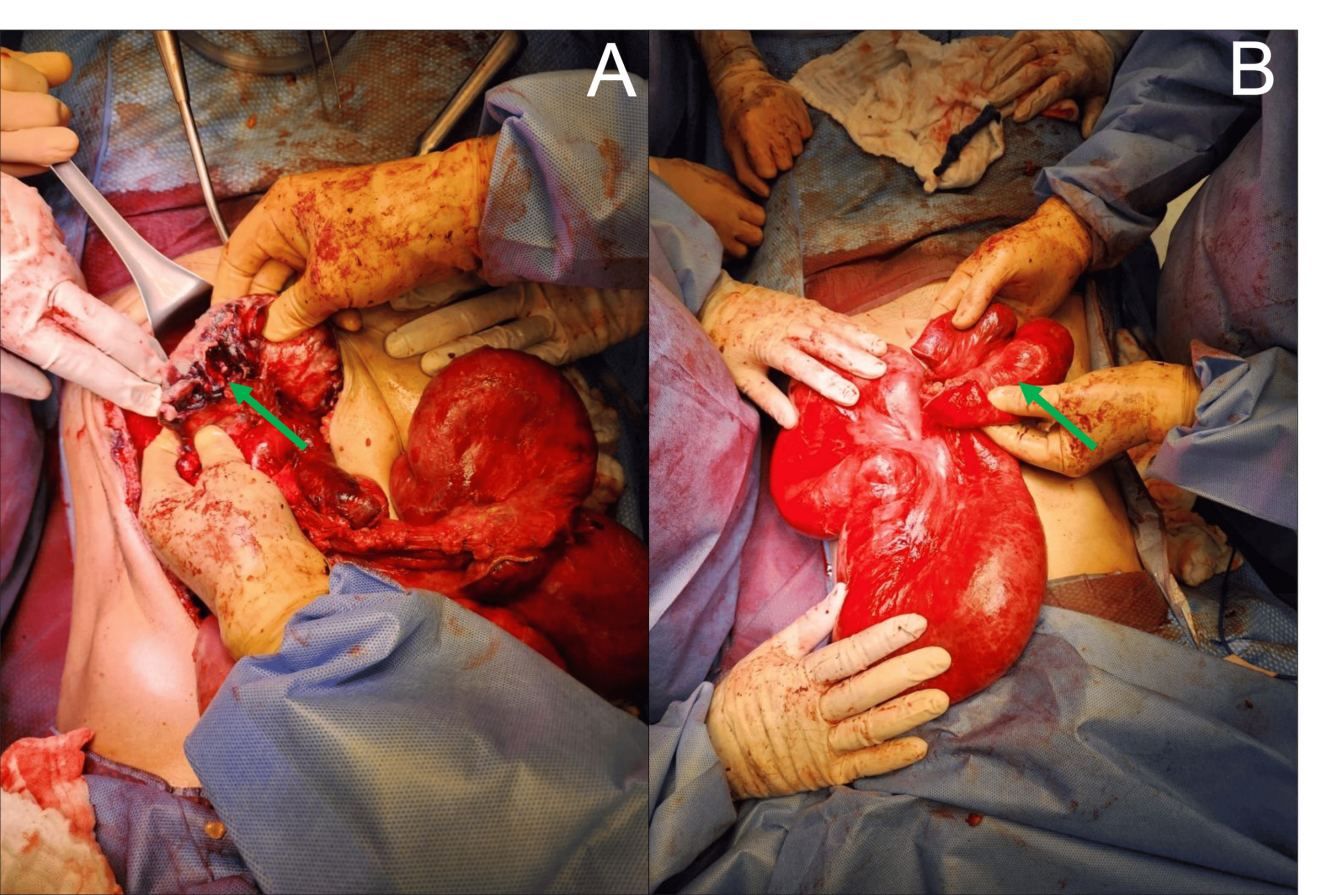
First laparotomy - Initial findings and segment of the resected ileum A. Inspection of the abdominal cavity during the first laparotomy revealed abdominal contamination, and central block of bowel loops with up to 12 cm of dilation, areas of ischemia, and three perforation sites (pointed in green) are noted. B. Segment of the ileum resection with notable small bowel dilation (pointed in green).

An exploratory laparotomy was performed, during which the patient developed septic shock, requiring dual vasopressor support and aggressive fluid resuscitation. Abdominal contamination was identified, along with firm adhesions between bowel loops and the abdominal wall, a central block of bowel loops with dilatation up to 12 cm, and obstruction at the ileum with areas of ischemia and three perforation sites (Figure 1A). Vascular congestion was noted. Adhesiolysis and abdominal lavage were performed, and approximately 20 cm of the ileum was resected using an 80 mm linear stapler (Figure 1B). The decision was made to defer anastomosis, leaving the bowel loops closed, and manage the open abdomen using a negative pressure system (Abthera™). Postoperatively, the patient was maintained with intermittent nasogastric suction (output between 300 and 500 mL/day) and received total parenteral nutrition.

**Figure 2:**
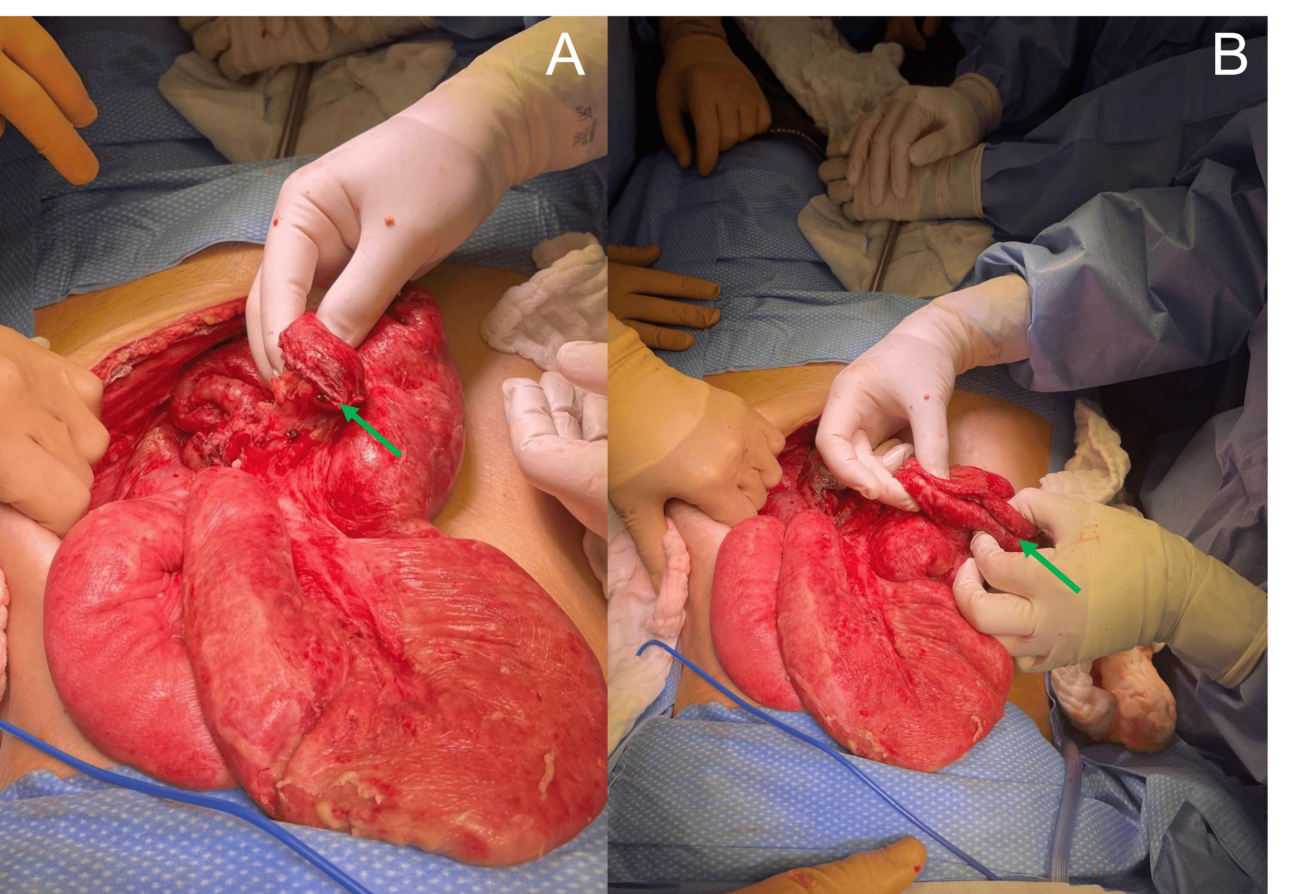
Second laparotomy - Closed bowel loops and inspection of the small bowel The second laparotomy revealed reduced edema and no signs of intestinal ischemia. A. closed bowel loops of the previously resected ileum (pointed in green) B. Clean abdominal cavity with reduced edema of the small bowel wall loops and no signs of ischemia.

A second surgery was performed 72 hours later, after discontinuation of vasopressor support and improvement in biochemical parameters. A clean abdominal cavity was observed, with reduced edema of the small bowel loops and no signs of ischemia (Figure 2A). After thorough inspection of the intestine (Figure 2B), the decision was made to defer anastomosis due to the patient's nutritional status, and the bowel loops were again closed, with replacement of the Abthera™ system for further lavage and re-evaluation.

**Figure 3:**
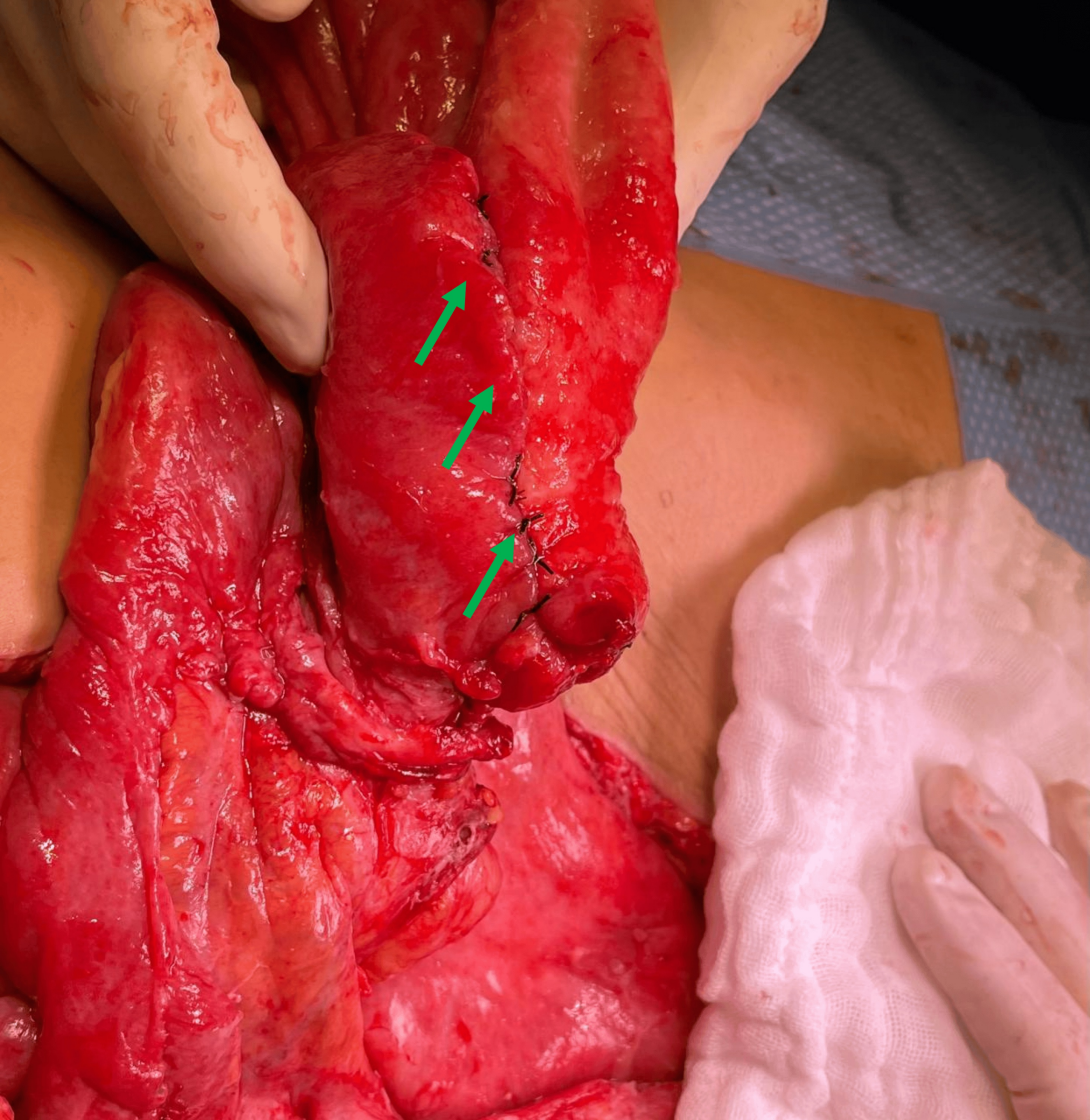
Third laparotomy - Anastomosis Side-to-side ileo-ileo mechanical anastomosis during the third laparotomy (pointed in green - three arrows following the sutures)

A third intervention was performed 140 hours after the initial laparotomy. No perforations were identified; however, persistent dilatation of a 20 cm segment of the jejunum was noted. This segment was resected, and a latero-lateral, antiperistaltic entero-enteric anastomosis was performed using an 80 mm linear stapler and polyglecaprone suture. The same technique was applied to the blind ends of the ileum. Fascial closure was achieved using long-absorption poly(4-hydroxybutyrate) sutures (Figure 3).

**Figure 4:**
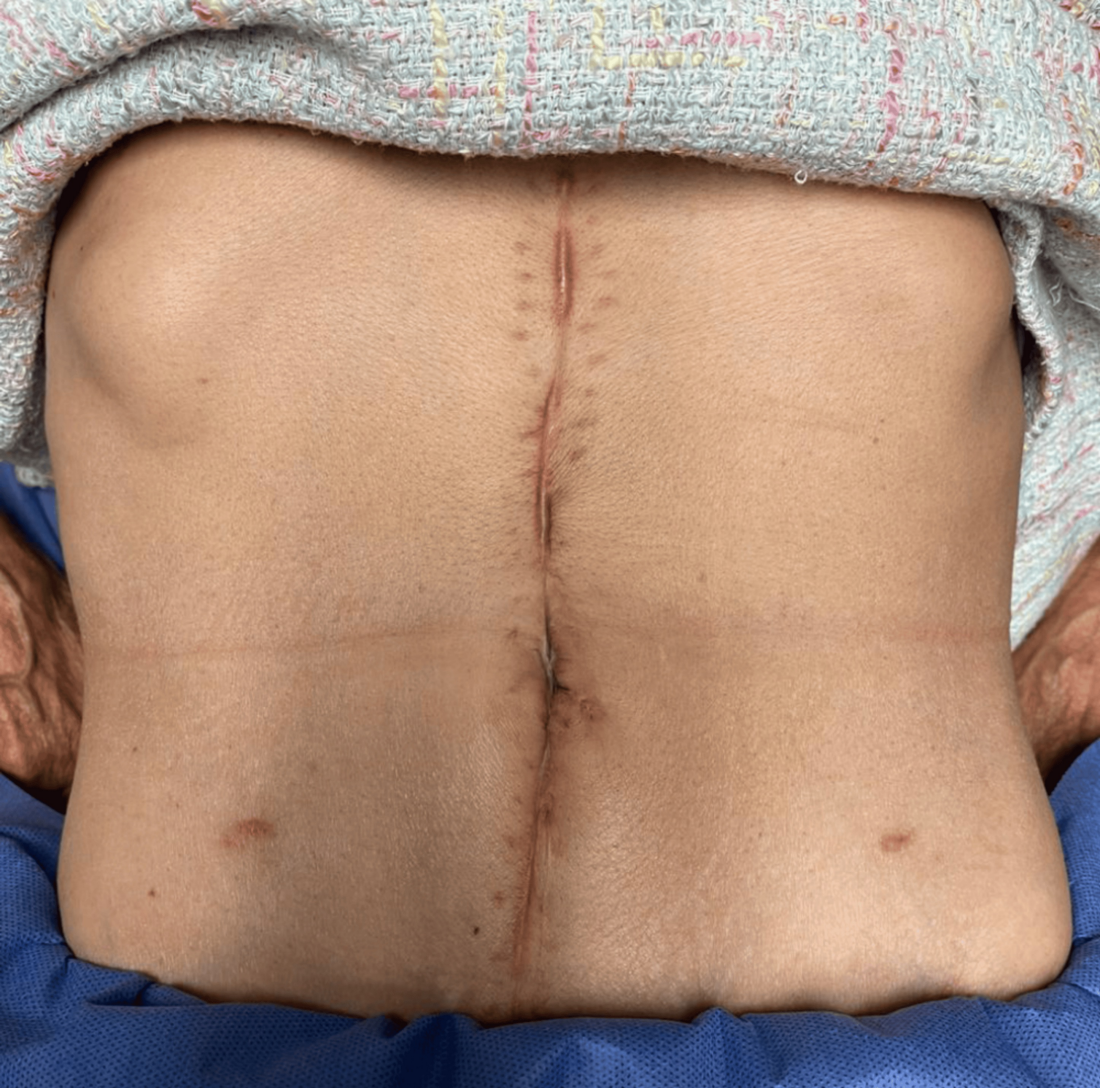
Follow-up - Inspection of the abdomen Patient's abdomen two months following initial surgery shows a clean scar with no complications.

The patient was discharged on postoperative day 10 following the final intervention, tolerating an oral diet and reporting no symptoms. Follow-up demonstrated significant improvement in nutritional parameters, with no complications (Figure 4).

## Discussion

DCS has been widely implemented in trauma settings. The World Journal of Emergency Surgery (WJES) published a guideline for managing non-traumatic patients, suggesting DCS as an option for patients with severe peritonitis associated with sepsis or septic shock under the following conditions: the need for re-evaluation of intestinal ischemia, failure to control the source of peritonitis, extensive visceral edema, or the risk of compartment syndrome [3]. This strategy allows for the correction of physiological abnormalities that place life at imminent risk, deferring definitive reconstruction until later. Factors improving survival probabilities with DCS have been identified, such as elevated lactate levels and multiple comorbidities [3,4].

The traditional dogma of managing hollow viscus perforations by creating stomas is unnecessary in some cases. Moreover, it may increase morbidity and the need for a second intervention to restore intestinal transit or leave a permanent stoma with the necessary care. In a retrospective study published by Mitchao et al. [5], 287 patients with colon injuries from a trauma center were included, of whom 101 required resections. Of these, 39.6% underwent damage control and temporary wall closure, 67% received primary anastomosis, 30% were managed with colonic discontinuity, and only 2.5% required a stoma. Primary anastomosis leakage was reported in only 8% of patients [5]. In the context of DCS, is it always feasible to perform an anastomosis, create a stoma, or defer it to a later stage to avoid prolonged interventions?

Traditionally, microcirculation and intestinal viability are evaluated subjectively by inspecting the serosal color, the presence of peristalsis, and bleeding from marginal arteries. The precision of the surgeon’s inspection to predict anastomotic leakage (AL) has been evaluated, with low predictive value [6,7]. The effectiveness of these techniques in reducing leakage rates by up to 60-84% in elective surgery patients has been demonstrated [8]. However, their utility in the context of DCS has not been reported. Other methods, such as those based on tissue oxygenation, Doppler-measured flow, mucosal pH, and fluorescence studies, have been evaluated. However, to date, no method meets the necessary criteria for routine use, nor has its applicability been confirmed in emergency surgery.

The procedure of temporary bowel loop ligation has been described in the literature as a maneuver in DCS for hemodynamically unstable patients [9]. A few studies compare the outcomes of traditional management, based on intestinal resections with primary anastomosis or intestinal diversion, with temporary bowel ligation.

During the initial exploration, the goal is to control intra-abdominal contamination and bleeding. If the patient is hemodynamically stable or responds to aggressive resuscitation, and after resecting the non-viable bowel segment, primary anastomosis may be considered. Otherwise, the objective is to shorten the surgical time. A valid option is placing a nasogastric tube for secretion management and gastric decompression, leaving the intestine in discontinuity, without definitive abdominal wall closure, to improve general parameters in the intensive care unit and later perform a revision surgery [10]. In the second laparotomy, 24-48 hours later, once the patient is stable and in better condition, the intestine should be inspected before restoring intestinal transit or, if necessary, creating a stoma. Studies in animal models subjected to hemorrhagic shock, multiple intestinal perforations, and hypothermia have shown that the temporary bowel ligation group required less resuscitation volume during resuscitation, achieved faster lactate clearance, and maintained greater hemodynamic stability [11].

In neonates with necrotizing enterocolitis, the term "clip-and-drop" refers to the resection of non-viable hollow viscus, temporary bowel ligation, and deferred anastomosis or stoma creation. A retrospective study from a single center evaluated the 30-day mortality and long-term outcomes of patients who underwent this procedure, presenting with hemodynamic instability, multifocal disease, significant intestinal involvement, and uncertain viability upon inspection. The reported mortality related to the intervention was 31.3%. As an advantage, only 9.1% developed short bowel syndrome, compared to other techniques, demonstrating that this strategy can preserve intestinal length [12].

Other studies contradict this practice, advocating for primary anastomosis even in cases of abdominal contamination, associating intestinal discontinuity with a higher risk of AL in a second surgical intervention [13]. Variables influencing these outcomes have been identified. A study on the management of small bowel injuries reported a higher leakage rate in cases of deferred anastomosis (with or without stoma) and a higher risk with more days of open abdomen [14]. Another factor to consider is a higher risk of intestinal ischemia compared to patients with primary anastomosis, associated with intestinal obstruction that causes bowel dilatation, bacterial translocation, mucosal damage, and the patient's hemodynamic state, which perpetuates hypoperfusion. This condition potentially increases the risk of AL due to the release of inflammatory mediators, which perpetuate organ failure. In a retrospective study published by Talving et al. [15], including patients from three trauma centers who required intestinal resection and damage control, a higher rate of intestinal ischemia was reported, adjusted for predictors (injury mechanism, age, injury severity, transfusion requirement): 13.1% in the anastomosed group vs 32.5% in the discontinuous group (p = 0.034). The infection and AL rates were similar in both groups [15].

In a study conducted at a level I trauma center, published by Ordoñez et al. [16], it was shown that 89.1% of patients undergoing DCS due to penetrating hollow viscus injuries successfully underwent deferred anastomosis. Only 8.2% required a stoma [16].

In a retrospective study analyzing the management of traumatic colon perforation with DCS, in which anastomosis was deferred in 30 patients with temporary closure of the colon until bleeding control, abdominal contamination, and abdominal wall management with a negative pressure system, a delayed anastomosis was performed in a second or third surgical intervention. They concluded that it is a safe alternative, with an 84% success rate for patients without persistent severe acidosis, significant intestinal wall edema, or intra-abdominal infections. Otherwise, a colostomy is suggested [5].

The management of the open abdomen has evolved considerably in recent years. Recently, the use of the negative pressure system has increased, and it is considered the gold standard to prevent the evolving complications of the open abdomen [11,13,14]. In addition to preventing frequent complications of an open abdomen, facilitating fascial closure, and providing protection for the viscera from the environment, the negative pressure system has been shown to reduce intestinal wall edema, improving conditions for a future anastomosis.

The dynamic laboratory profile (Table 1) supports the clinical course, with an initial systemic inflammatory response syndrome (SIRS) pattern characterized by leukocytosis and markedly elevated C-reactive protein levels. These gradually improved following surgical source control and antibiotic therapy, consistent with the resolution of intra-abdominal infection. The drop in hemoglobin was attributed to surgical blood loss and dilutional effects due to fluid resuscitation. Albumin levels, though persistently low, improved slightly toward discharge, reflecting a slowly recovering nutritional and inflammatory state.

Many factors can confound the results of implementing this technique. Patients undergoing DCS are severely ill, with comorbidities and a high risk of morbidity. The increased preference for performing primary anastomosis even in scenarios of abdominal contamination and hemodynamic instability has reduced the use of this technique. There are no clear guidelines on temporary bowel loop ligation as a rescue maneuver, including indications, the time allowed before definitive repair, how to manage such patients, and the impact on the leakage of deferred anastomosis.

## Conclusions

This case report describes the successful management of complex abdominal-origin sepsis in a 64-year-old patient with multiple comorbidities and a history of cancer, through the combination of temporary bowel loop ligation and NPT. The damage control surgery strategy, including temporary bowel loop ligation in the first intervention and subsequent delayed anastomosis, allowed for the hemodynamic stabilization of the patient and minimized the risk of complications in the context of initial instability and malnutrition. The use of NPT in the open abdomen facilitated the control of contamination, reduced intestinal edema, and prepared the ground for a safe anastomosis in a later intervention. The decision to delay the anastomosis, although controversial, proved to be a successful strategy in this case, avoiding the need for a stoma and allowing for the successful restoration of intestinal continuity.

While the literature presents mixed results regarding temporary bowel loop ligation, this case demonstrates its potential in critical situations. Further studies are needed to establish clear protocols and define the selection criteria for patients who would benefit from this technique. The experience presented here contributes to the knowledge on the management of complex intestinal perforations and highlights the need to individualize treatment according to the characteristics of each patient.
